# The effects of anthocyanin-rich wheat diet on the oxidative status and behavior of rats

**DOI:** 10.3325/cmj.2016.57.119

**Published:** 2016-04

**Authors:** Katarína Janšáková, Janka Bábíčková, Michaela Havrlentová, Július Hodosy, Ján Kraic, Peter Celec, Ľubomíra Tóthová

**Affiliations:** 1 Institute of Molecular Biomedicine, Faculty of Medicine, Comenius University, Bratislava, Slovakia; 2 Institute for Clinical and Translational Research, Biomedical Research Center, Slovak Academy of Sciences, Bratislava, Slovakia; 3 Research Institute of Plant Production, Piešťany, Slovakia; 4 Department of Biotechnology, Faculty of Natural Sciences, University of SS. Cyril and Methodius, Trnava, Slovakia; 5 Institute of Physiology, Faculty of Medicine, Comenius University, Bratislava, Slovakia; 6 Institute of Pathophysiology, Faculty of Medicine, Comenius University, Bratislava, Slovakia; 7 Department of Molecular Biology, Faculty of Natural Sciences, Comenius University, Bratislava, Slovakia

## Abstract

**Aim:**

To evaluate the effect of food containing anthocyanin-rich wheat on oxidative status and behavior of healthy rats.

**Methods:**

Twenty male rats were divided into the control and anthocyanin group. Oral glucose tolerance test was performed, and proteinuria and creatinine clearance were measured. Behavioral analysis was performed in Phenotyper cages. Serum and tissues were collected to measure the markers of oxidative stress and antioxidant status.

**Results:**

Anthocyanins significantly increased total antioxidant capacity in serum (*P* = 0.039), decreased advanced oxidation protein products in the kidney (*P* = 0.002), but increased thiobarbituric acid reactive substances in the kidney compared to the control group. No significant difference between the groups was found in the markers of oxidative stress in the liver and colon, as well as in renal functions and glucose metabolism. The anthocyanin group spent significantly less time in the spotlight zone of the Phenotyper cages (*P* = 0.040), indicating higher anxiety-like behavior.

**Conclusion:**

Food containing anthocyanin-rich wheat had positive effects on serum antioxidant status and kidney protein oxidation, but increased lipid peroxidation in the kidney and modified animal behavior related to anxiety. The molecular mechanisms leading to observed effects should be further elucidated.

Antioxidants prevent the generation of free radicals, thus decreasing oxidative damage to macromolecules. They are formed endogenously in the organism or are obtained exogenously from the food (1). Although, it is generally accepted that increased antioxidant intake leads to decreased plasma markers of protein and lipid peroxidation, studies found contradictory results. The intake of vitamin C in drinking water (0.5 mg/mL) did not affect the concentration of plasma markers of protein and lipid peroxidation in healthy rats compared to the control group (2). On the other hand, supplementation with α-tocopheryl acetate in healthy middle-aged and elderly people significantly increased the plasma concentrations of antioxidant enzyme, superoxide dismutase, and decreased the concentration of lipid peroxidation marker, malondialdehyde, compared to the baseline concentrations (3).

Nowadays, there is an increased interest in flavonoids, especially anthocyanins. Anthocyanins are plant water soluble pigments that can be found in fruit (eg, grapes, blackcurrant), vegetables (eg, red cabbage), petals of ornamental plants (4-6), and whole grain cereals, including wheat (7). They appear transiently at specific stages of plant development, depending on a number of environmental factors, such as visible and UV-B radiation, high or low temperatures, or drought (8).

Both human and animal studies have shown that anthocyanins have an anti-inflammatory and cardioprotective effect and inhibit oxidative stress by scavenging free radicals (9-11). Acute anthocyanin-rich juice intake decreased the serum levels of thiobarbituric acid reactive substances (TBARS) compared to the baseline levels (12). Similar results were observed in the serum of healthy volunteers after 30 days of strawberry intake (13). Another study showed that the size of myocardial infarction induced in both *in vivo* and *ex vivo* models was significantly reduced in the groups fed with anthocyanin-rich food (for 8 weeks) compared to the control group (13). Similarly, dietary flavonoids intake was associated with lower risk of type 2 diabetes mellitus in humans (14) and improved insulin resistance and blood glucose levels in rats (15).

Reactive oxygen species (ROS) include free radicals and non-radical molecules are formed during physiological processes (16). They play an important role in energy metabolism and are also involved in immune responses (17,18). However, increased production of free radicals, decreased activity of antioxidant mechanisms, or both, cause oxidative stress (19,20). Oxidative stress is closely related to carbonyl stress, which is associated with the production of advanced glycation end products (AGEs). The accumulation of free radicals causes oxidative damage to cell components, such as DNA, lipids, and proteins (21,22). The inability of cellular repair mechanisms to cope with extensive damage can lead to cell apoptosis or necrosis (16).

While it is believed that anthocyanins naturally occurring in food might be beneficial for cardiovascular diseases (23) or cognitive functions (24), it is not clear whether these beneficial effects are produced by anthocyanins themselves or by food complexity. Therefore, the aim of this study was to describe the metabolic effect of anthocyanins-rich wheat, since wheat itself is not rich in anthocyanins. Additionally, the effect of anthocyanins on the brain functions was investigated by behavior monitoring.

## Materials and methods

### Experimental design

All experiments were performed in full compliance with the EU Guidelines for Scientific Experimentation on Animals and the study was approved by the Ethics Committee of the Institute of Molecular Biomedicine, Faculty of Medicine, Comenius University under number 05/2013/SKP1012 on 29.1.2013. The study was conducted in the period 2013-2014, and the wheat for pellets was composed from seeds improved and tested a few years before.

**Figure 1:**
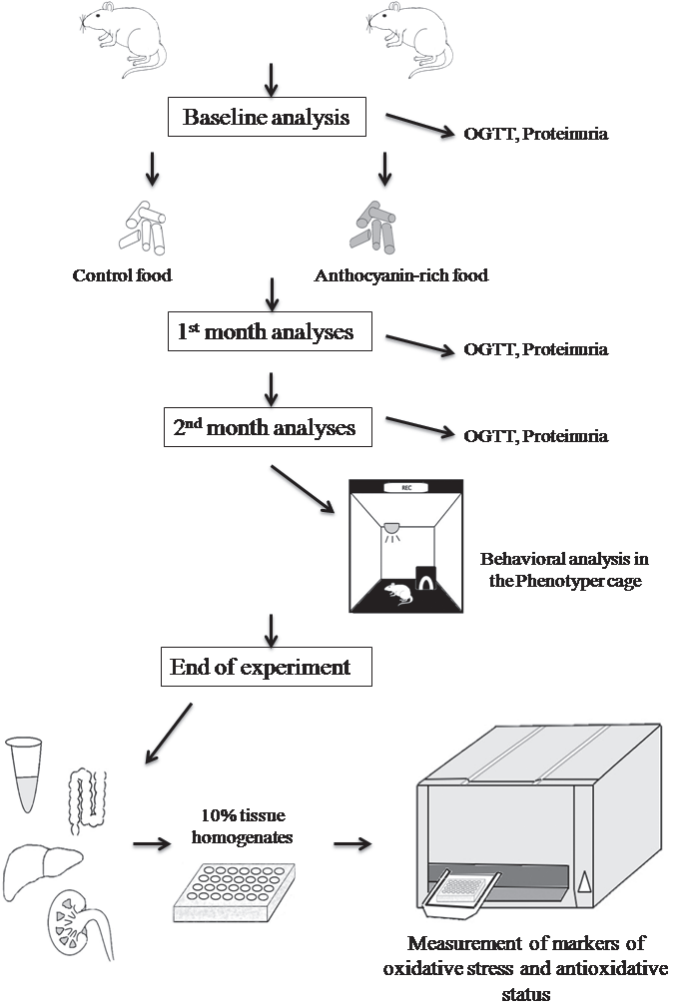
Experiment design.

Twenty male Wistar rats (28 days old) were purchased from Anlab (Prague, Czech Republic). All rats were kept separately under 12 hours light/dark cycle. Rats were assigned into one of two groups (10 per group) by tossing a coin. The anthocyanin group received food enriched with anthocyanins for 2 months. The control group received control diet for the same time period. The feeding started on the 28th postnatal day. Both groups received water and food *ad libitum*. The weight of rats and food consumption was monitored once per week. At the end of the experiment, animals were anesthetized using intraperitoneal ketamine and xylazine (100 mg/kg and 10 mg/kg, respectively), and blood samples were collected from the abdominal aorta into serum tubes (Sartedt, Numbrecht, Germany) (Figure 1).

### Diet

Two types of food pellets were used. The control group was fed a diet prepared from the seeds of wheat (*Triticum aestivum* L.) cultivar Viglanka without other additives. The experimental group was fed the control diet with addition of 20.5% of whole wheat seeds of the cultivar Karkulka, rich in anthocyanin (the total anthocyanins content 110 mg/kg dwb). The content of anthocyanins was assed according to Fuleki and Francis (25). Briefly, the pH of anthocyanin-rich extracted solution was adjusted to 1.0 and 4.5. Thereafter, the absorbance was measured at 520 nm and 700 nm. The concentration of anthocyanins was calculated to cyaniding-3-glucoside equivalents. Both food types were prepared in The Plant Production Research Center, Piešťany, Slovakia. Roughly milled whole wheat seeds were moistened by distilled water to the humidity of 16% and then pressed at laboratory temperature to pellets with a diameter ±10 mm.

### Sample preparation

Blood samples were collected into Serum collecting tubes (Sarstedt, Numbrecht, Germany) from the aorta at the end of the experiment. After centrifugation (2000 g for 7 min), serum was immediately frozen at -20°C. 24-hour urine was collected once per month and stored at -20°C. The colon, liver, and kidney tissues were homogenized in phosphate-buffered saline in order to prepare 10% homogenates (PBS, pH = 7.2). After homogenization, the samples were centrifuged at 4000 g for 10 min. The supernatant was collected and stored at -20°C until further analysis.

### Biochemical analysis

Markers of oxidative and carbonyl stress and antioxidant status were measured in the serum and colon, liver, and kidney homogenates. Markers of protein oxidation were measured by spectrophotometric analysis of advanced oxidation protein products (AOPP). In this assay, 200 µL of samples (diluted 1:4 in PBS, pH = 7.2) were mixed with 20 µL of glacial acetic acid. For the calibration curve construction, chloramine T with potassium iodide was used. The absorbance was measured at 340 nm (26).

Markers of lipid peroxidation, TBARS, were measured by spectrofluorometric method. 20 µL of samples were mixed with 20 µL of thiobarbituric acid, 20 µL of glacial acetic acid, and 30 µL of water. Thereafter, samples were shortly centrifuged and incubated at 95°C for 45 min. After incubation, samples were derivatized with n-butanol and centrifuged at 2000 g and 4°C for 10 min. The upper phase was transferred into the microtiter plate and measured at λex. = 515 nm and λem. = 535 nm (27). As the standard for construction of the calibration curve, 1,1,3,3 tetraethoxypropane was used.

The following markers of carbonyl stress were measured: AGEs and fructosamine. AGEs were measured spectrofluorometrically (28). 20 µL of samples were diluted with PBS (pH = 7.2). Fluorescence was measured at λex. = 370 nm and λem. = 440 nm. AGE-modified bovine serum albumin was used as standard in the calibration curve. For fructosamine measurement, 20 µL of samples and standards (1-deoxy-morpholino-D-fructose) were added to the microtiter plate. Thereafter, nitro blue tetrazolium was added, and the reaction was shortly mixed and incubated at 37°C for 15 minutes. The absorbance was measured at 530 nm (29).

The following markers of antioxidant status were measured: Total antioxidant capacity (TAC) and ferric reducing antioxidant power (FRAP). TAC was measured according to Erel et al (30). The samples were mixed with acetate buffer (pH = 5.8). The initial absorbance was measured at 660 nm as blank. When ABTS solution (2.2'-azino-bis(3-ethylbenzthiazoline-6-sulphonic acid with acetate buffer) was added, the absorbance (660 nm) was measured again. FRAP was measured after the addition of FRAP reagent (warmed to 37°C, composed of acetate buffer (pH = 3.6), tripyridyl-*s*-triazine, FeCl_3_ × 6H_2_O and water) to the microtiter plate. Afterwards, initial absorbance was measured as blank. The samples were added to reagent and measured again at 593 nm (31). The concentration of proteins was measured by bicinchoninic acid kit (Sigma-Aldrich, Munich, Germany), according to the manufacturer’s instructions. The bovine serum albumin was used as standard. All measurements were performed on a Tecan Sapphire II instrument (Grödig, Austria). All reagents used in this study were obtained from Sigma-Aldrich (Munich, Germany).

### Glucose metabolism

Oral glucose tolerance test was performed on overnight fasting rats at the beginning of the experiment and monthly during the experiment. Glucose was administered orally by gastric gavage (2 g/kg of body weight in 500 µL of water) (32). Glycemia was measured at multiple time intervals (0, 15, 30, 60, 90, and 120 min) with an automatic glucometer Accu-chek Performa (Roche, Basel, Switzerland).

### Renal metabolism

Urinary creatinine was measured using the spectrophotometric method by Jaffe (33). 10 µL of samples were mixed with mixture of NaOH and picric acid (ratio 5:1). After designed time, the absorbance was measured at 492 nm. Serum creatinine concentrations were measured using a commercial creatinine serum detection kit (Arbor Assays, Ann Arbor, MI, USA) according to the manufacturer’s instructions. Briefly, 15 µL of samples were mixed with 15 µL of assay diluent and 60 µL of creatinine reagent. The absorbance was measured at 492 nm. The creatinine clearance was determined as follows:


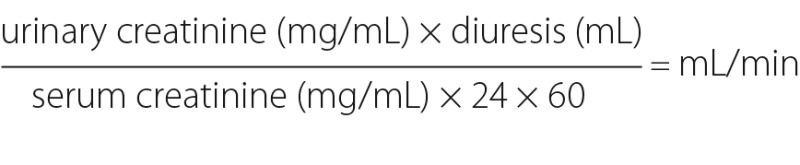


Proteinuria was analyzed by the pyrogallol red method (34). 10 µL of samples were mixed with 300 µL of pyrogallol red reagent (composed from pyrogallol red, methanol, succinic acid, sodium oxalate, sodium benzoate, disodium molybdate, sodium dodecyl sulfate, and Brij 35). After incubation at 37°C for 15 minutes, the absorbance at 660 nm was measured. Bovine serum albumin was used to construct the calibration curve.

### Behavioral phenotyping

**Figure 2:**
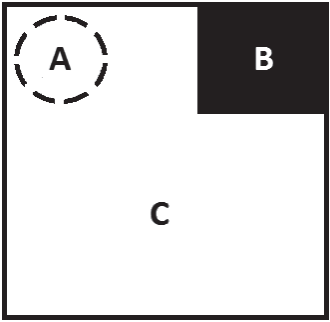
Zone distribution in the Phenotyper cage: (**A**) spot light zone, (**B**) shelter zone, (**C**) open space as well as dark zone of the maze when the spot light is turned on.

One week before the end of the experiment, behavioral analysis was performed using Ethovision XT 10 tracking software (Noldus, Wageningen, Netherlands) for 24 hours in special instrumented observation cages – Phenotypers, serving as home cages (Noldus Information Technology, *www.noldus.com/phenotyper*). The food and water were available *ad libitum*. The instrumented cage consists of 45 × 45 cm black base floor and a top unit with an integrated infrared sensitive camera and a source of a white spot light. Inside the cage, a black shelter was placed to one of the corners with 2 distinct entrance holes. The spot light was turned on for 6 hours between 10:00 pm to 04:00 am The base of the floor of the instrumented cage was virtually divided by Ethovision software into the shelter and open zone. The open zone was further divided into a spotlight zone and a dark zone (Figure 2). The transitions between the zones, distance moved, and average speed were analyzed to assess general locomotor activity. The time spent in the shelter and the open zones, especially the spotlight zone, were analyzed to assess the anxiety level.

### Statistical analysis

Data are presented as the mean ± standard deviation (SD). All parameters were compared using unpaired two-tailed *t* test and repeated measures 2-way ANOVA after testing for normality by Shapiro-Wilk test. Consequently, a Sidak test was used for post-hoc correction. For evaluation of behavior and comparison of time spent in specific zones and frequencies, a 2-way ANOVA was used, with one factor being the zone and the second factor being the treatment. Statistical significance level was set to *P* = 0.05. The results were analyzed using GraphPad Prism 5.0 (GraphPad Software, Inc., La Jolla, CA, USA).

## Results

**Figure 3:**
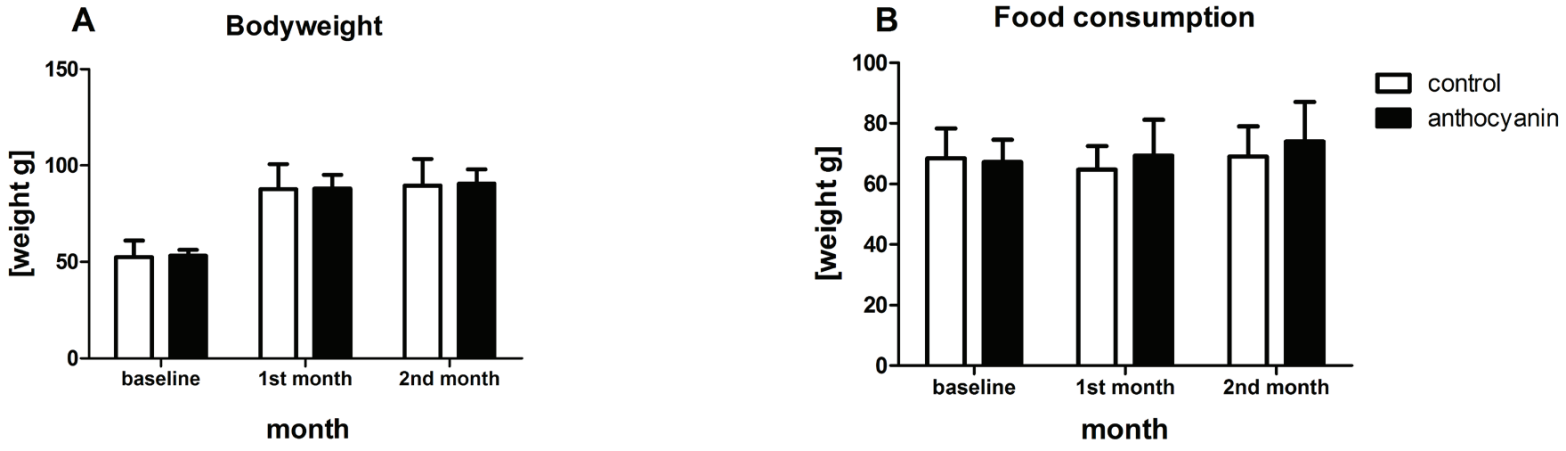
No significant difference between the control and anthocyanin group was found in (**A**) bodyweight and (**B**) food consumption. Data are presented as mean ± standard deviation.

**Figure 4:**
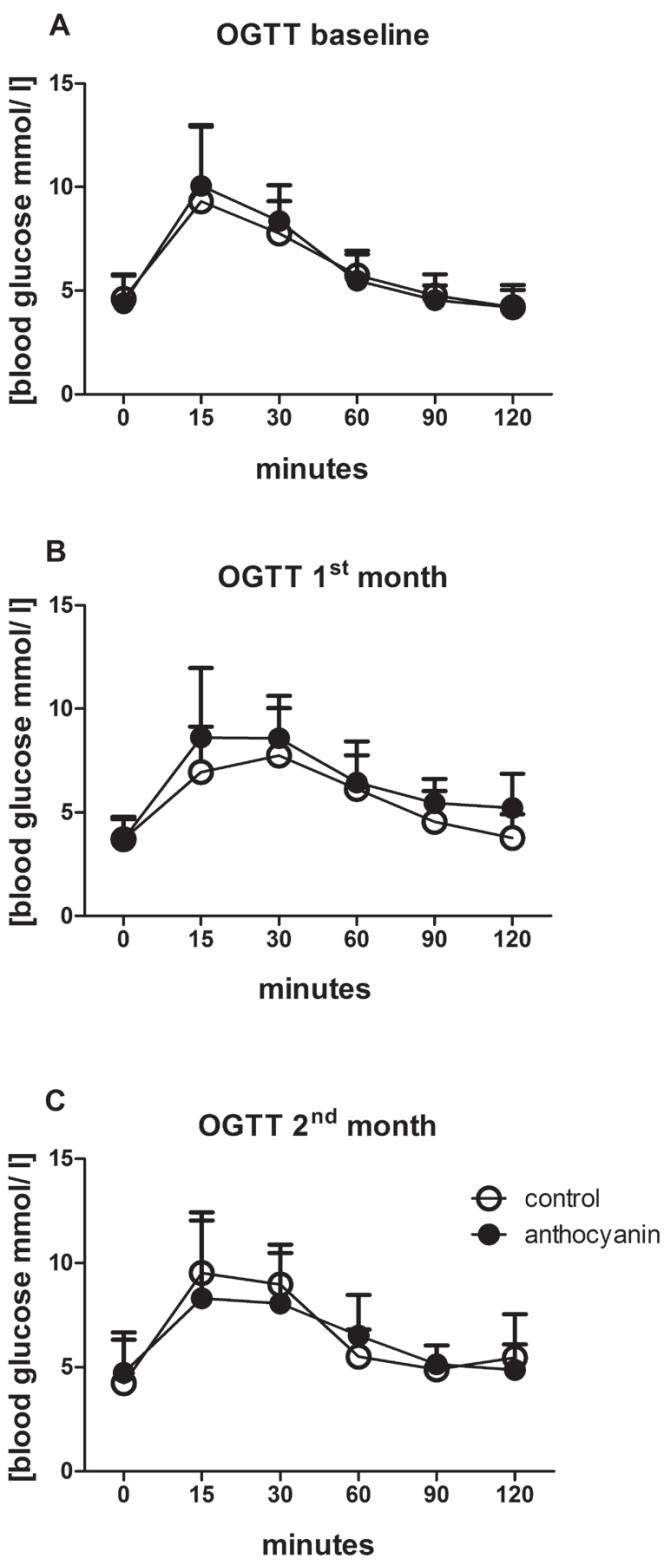
No significant difference between the control and anthocyanin group was found in glycemia during oral glucose tolerance test at (**A**) baseline, (**B**) at 1 month, (**C**) and at 2 months. Data are presented as the mean ± standard deviation.

**Figure 5:**
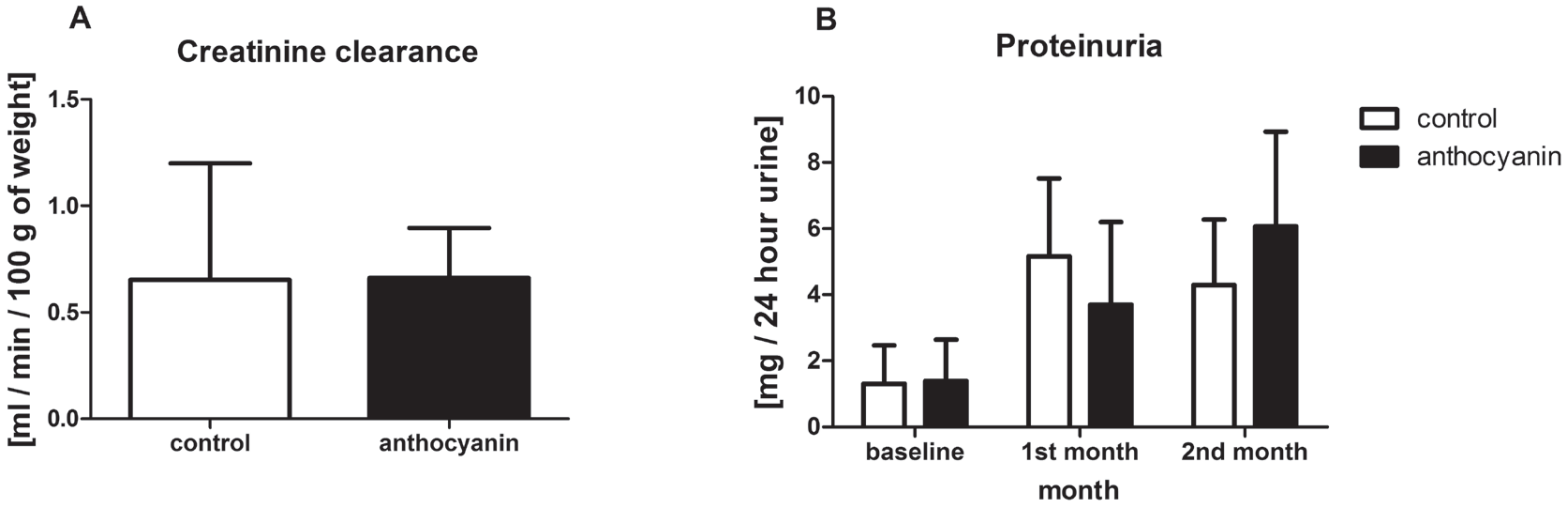
No significant difference between the control and anthocyanin group was found in (**A**) creatinine clearance analyzed at 2 months and (**B**) proteinuria analyzed at baseline, at 1 month, and at 2 months. Data are presented as mean ± standard deviation.

There were no significant differences in bodyweight and food consumption between the anthocyanin and control group (Figure 3). Food consumption did not differ between the analyzed months. The groups did not significantly differ in the oral glucose tolerance test results at baseline, at 1 month, and at 2 months (Figure 4). In the control and anthocyanin group, the highest concentration of blood glucose was observed 15 minutes after glucose administration at baseline (by 202% and 229% compared to 0 min, respectively), at 1 month (by 187.6% and 233% compared to 0 min, respectively), and at 2 months (by 225% and 175.7% compared to 0 min, respectively). The blood glucose returned back to normal values within 90 minutes in both groups. Proteinuria and creatinine clearance did not differ significantly between the groups (Figure 5). When compared to the baseline values, both the control and anthocyanin group showed higher proteinuria at 2 months (by 329% and 436%, respectively).

**Figure 6:**
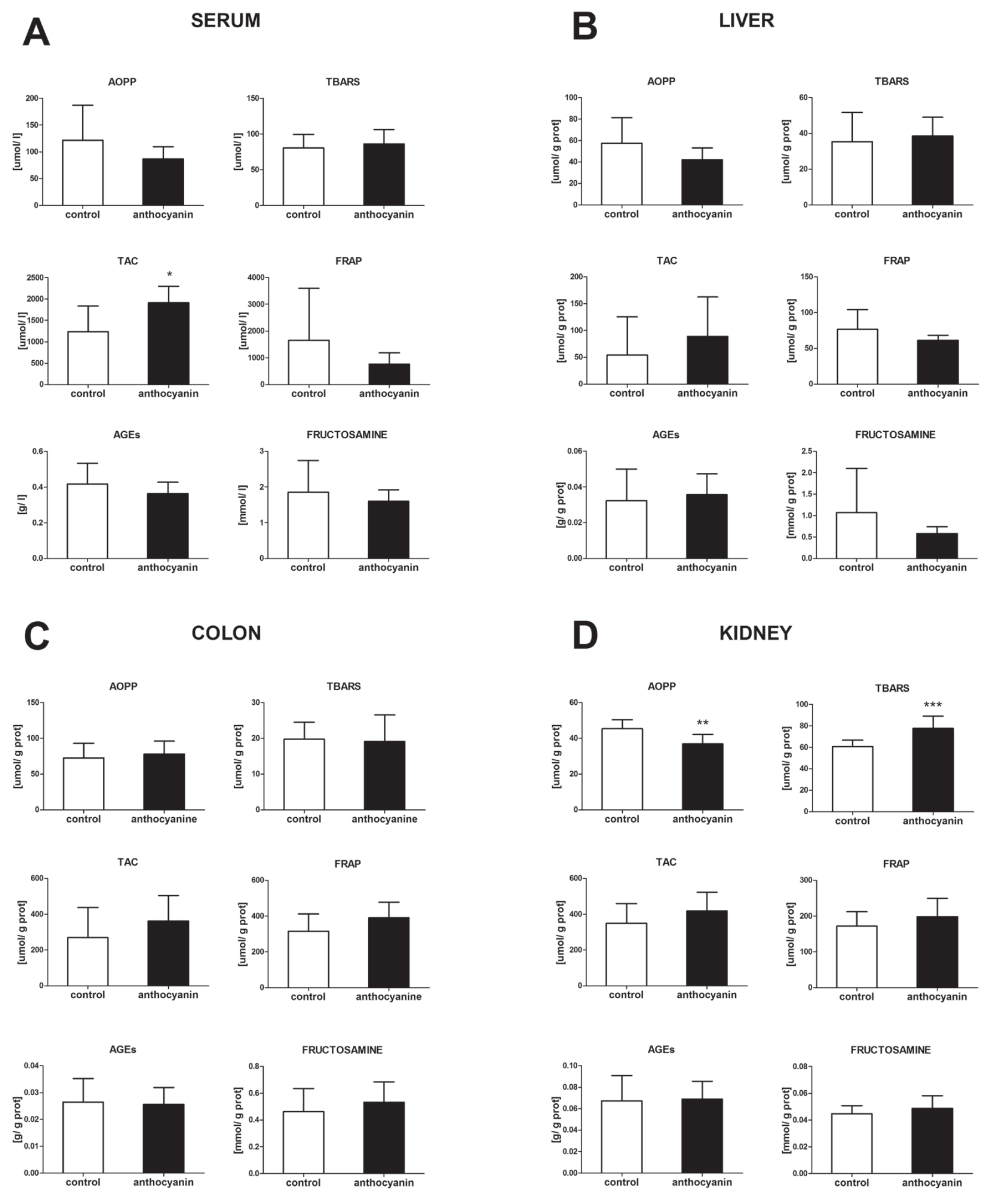
Difference between the control and anthocyanin group in markers of oxidative, carbonyl stress, and antioxidant status measured in the (**A**) serum, (**B**) liver, (**C**) colon, and (**D**) kidney homogenates. AOPP – advanced oxidation protein products, TBARS – thiobarbituric acid reactive substances, TAC – total antioxidant capacity, FRAP – ferric reducing antioxidant power, AGEs – advanced glycation end products.*P < 0.05, **P < 0.001, ***P < 0.001. Data are presented as mean ± standard deviation.

In the anthocyanin group, serum AOPP (Figure 6A) were lower by 28.5%, serum TBARS were higher by 7%, and serum AGEs and fructosamine were lower by 13% and 14%, respectively, compared to the control group but none of these differences reached significance. TAC was significantly higher in the anthocyanin group by 53.8% compared to the control group (*P* = 0.039). FRAP concentration in the anthocyanin group was lower by 53.7% compared to control group, but the difference was not significant.

In the liver (Figure 6B) and colon (Figure 6C), none of the measured markers showed any significant differences between the analyzed groups. In the kidney (Figure 6D), AOPP were significantly lower in the anthocyanin group by 18.8% compared to the control group (*P* = 0.002), but TBARS levels were significantly higher by 27.3% (*P* = 0.001).

**Figure 7:**
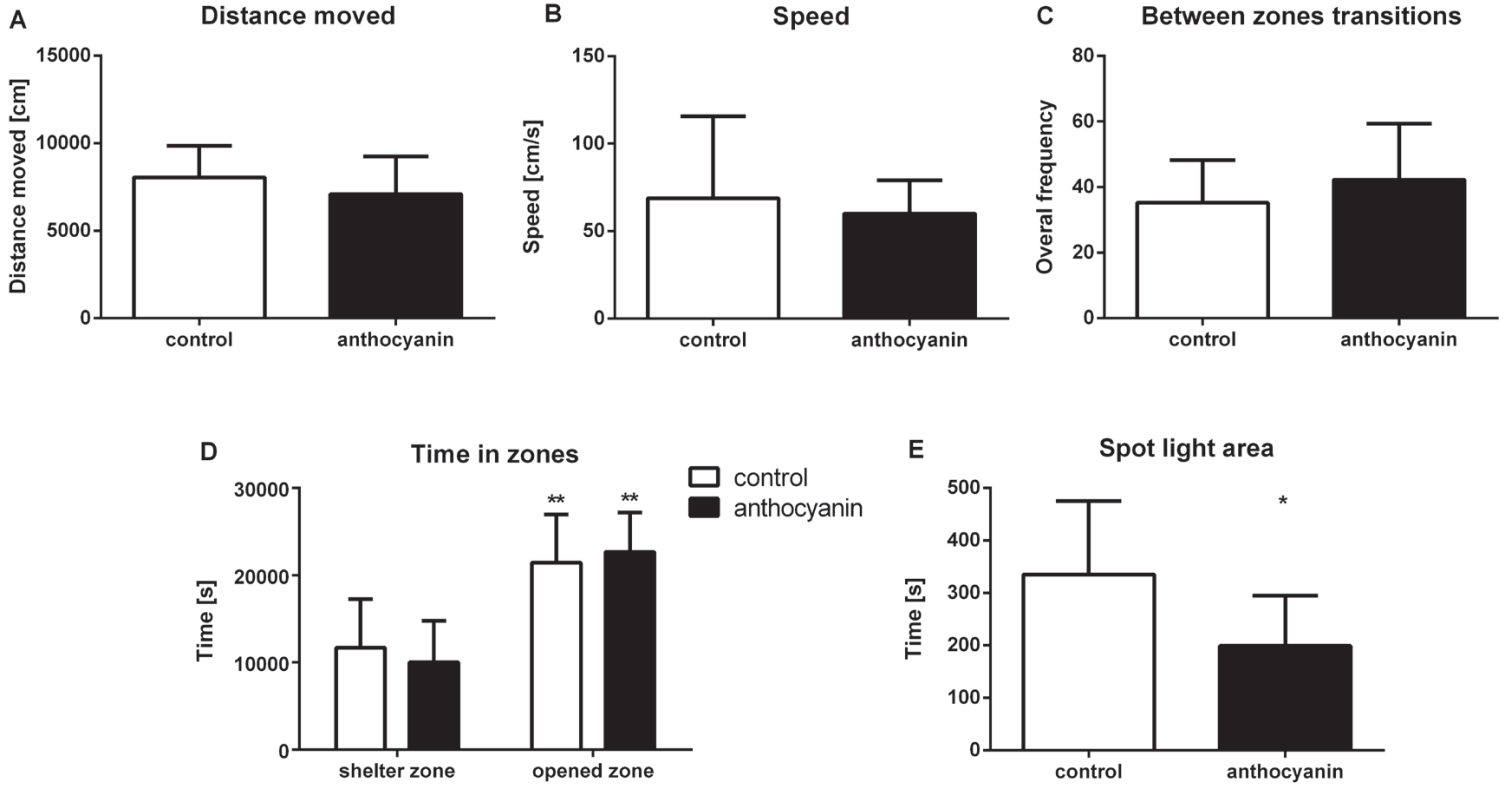
Difference between the control and anthocyanin group in general motor activity, expressed as (**A**) overall distance covered in open zones, (**B**) average speed achieved in open space, (**C**) zone transitions, (**D**) time spent in shelter or open zones, and (**E**) time spent in a spot light area of open zone; *P < 0.05 compared to the control group, **P < 0.01 compared to control shelter zone. Data are presented as the mean ± standard deviation.

Behavioral analysis revealed no significant differences between the analyzed groups in overall distance covered, average speed, and zone transitions (Figure 7A, 7B and 7C, respectively). ANOVA showed the zone factor, but not the groups, to be a significant determinant of the time spent. The animals from both groups spent significantly more time in the open zones than in the shelter zone (Figure 7D; *P* = 0.004 and *P* = 0.001 for the control and anthocyanin group, respectively). Although no difference between the groups was found in the time spent in the open zones, the anthocyanin group spent significantly less time in the spotlight zone than the control group (Figure 7E; *P* = 0.040).

## Discussion

The main finding of this study was the significantly higher total antioxidant status in the serum of rats fed with the anthocyanin-rich food than in the control group observed after 2 months of anthocyanin-rich food intake. Similar results were observed by Hassimotto et al (35), who found increased antioxidant capacity in the plasma of rats after 35 days intake of anthocyanin-rich fraction from blackberries. Significantly higher plasma antioxidant status was also reported in rats after 12 weeks of vitamin E-deficient diet supplemented by anthocyanin-rich extract (1 g/kg diet) (36). However, Perez-Jimenez et al showed that 16 weeks of grape antioxidant dietary fiber intake (7.5 g of powder per day) had no significant effect on total antioxidant capacity (37). This discrepancy could be explained by the fact that they did not consider other antioxidants intake as an exclusion criterion.

In the present study, the measurement of oxidative stress markers revealed contradictory results. Serum, liver, and kidney samples in the anthocyanin group had higher concentration of lipid peroxidation markers but lower concentration of protein oxidation markers than the control group, and a significant difference was observed only in the kidney samples. These results are inconsistent with the results by García-Alonso et al (12). In their study on the oxidative status of healthy volunteers, acute intake of anthocyanins-enriched juice increased the concentration of protein oxidation markers, but decreased lipid peroxidation markers in the plasma already after one hour of intake (12).

We observed a lower concentration of markers of lipid peroxidation and the same level of markers of protein oxidation in the colon homogenates of both groups. The analysis of glucose metabolism revealed no difference between the groups. Another study has reported that the intake of anthocyanin-rich fat-free soybean flour decreases blood glucose in obese and hyperglycemic mice during 13 weeks of blueberry anthocyanin treatment (38). In the present study, anthocyanin-rich food was administered to normoglycemic rats. Probably, anthocyanin-rich food has beneficial effects only on elevated glucose levels, while the physiological concentrations of glucose remain unaffected. This assumption needs to be verified in further studies focusing on the effect of anthocyanin-rich diet on glucose metabolism in various models of metabolic disorders.

Behavioral analyses in the Phenotyper cages revealed no differences in general locomotor activity between the control and anthocyanin group. This finding is similar to the study by Tall et al (9), which showed that high dose of anthocyanin (400 mg/kg of rat) had no effect on the motor functions analyzed during accelerating rotarod experiment. However, the anthocyanin group surprisingly showed higher anxiety level, expressed as decreased time under the bright white light. Contrary to these findings, Rabbani et al (39) found that *Stachys levandulifolia* extract decreased the anxiety level in mice observed through the elevated plus maze testing. They observed a similar result also after treatment with hydroalcoholic extract of *Salvia reuterana* (40). Several other studies showed that anthocyanin-rich food improved memory and had anxiolytic effect (41,42). Additionally, other studies reported that anthocyanins prevented memory impairment and decreased the anxiety level in rats with induced sporadic dementia of Alzheimer type (43). In light of these other studies, induction of anxiety-like behavior by anthocyanins in our study cannot be fully explained. However, this is the first animal study that observed behavioral changes during 24-hour monitoring in instrumented observational cages after long-term regular intake of anthocyanin-rich food. Since free radicals play a significant role as second messengers and in communication between the cells, it is possible that long-term increase in antioxidant capacity and decreased free radical formation lead also to higher anxiety level through impaired cell signaling. Nevertheless, this hypothesis should be further tested using molecular biology methods.

The major limitation of this study was the composition of food pellets. The feeding granules were composed only of pure control wheat (control group) or anthocyanin-rich wheat (anthocyanin group). The rats had no access to other food sources. However, the nutritional value of the wheat pellets is dubious. During the experiment relatively low food consumption was observed in both groups. We believe that future studies should use standard rat chow enriched with anthocyanins-rich wheat.

In conclusion, two months of consumption of the anthocyanin-rich food increased the antioxidant capacity in the serum and decreased protein oxidation in the kidney. To the best of our knowledge, this is the first animal study analyzing the effects of anthocyanin-rich food on metabolism, oxidative status, and behavior in healthy animals. Although mostly negative, the results are of importance as they show that the intake of anthocyanin-rich food does not induce toxic effects, at least in the analyzed tissues and on the analyzed functions. The observed higher lipid peroxidation in the kidney does not seem to have any functional consequences. Further long-term studies should prove these effects in animal models of diseases related to oxidative stress and inflammation.
